# Civic engagement and mental health system strengthening in Indonesia: a qualitative examination of the views of health professionals and national key stakeholders

**DOI:** 10.1186/s12888-020-02575-3

**Published:** 2020-04-15

**Authors:** Irman Irmansyah, Herni Susanti, Karen James, Karina Lovell, Sri Idaiani, Soimah Imah, Giur Hargiana, Budi-Anna Keliat, Bagus Utomo, Erminia Colucci, Helen Brooks

**Affiliations:** 1 National Institute of Health Research and Development, Jakarta, Indonesia; 2 Marzoeki Mahdi Hospital, Bogor, Indonesia; 3 Faculty of Nursing, Universitas Indonesia, Depok, Indonesia; 4 Centre for Health and Social Care Research, Faculty of Health, Social Care and Education, Kingston and St Georges, London, UK; 5 Division of Nursing, Midwifery and Social Work, School of Health Sciences, Faculty of Biology, Medicine and Health, University of Manchester, Manchester Academic Health Science Centre, Manchester, UK; 6 Greater Manchester Mental Health NHS Foundation Trust, Manchester, UK; 7 KPSI, Jakarta, Indonesia; 8 Department of Psychology, Middlesex University, London, UK; 9 Department of Health Services Research, Institute of Population Health Sciences, University of Liverpool, Room B112, Waterhouse Building Block B, Liverpool, UK

**Keywords:** Civic engagement, Implementation, Indonesia, Low- and middle-income countries, Mental health, Patient and public involvement

## Abstract

**Background:**

Mental health services in Indonesia are developing rapidly in response to national and global health policy to support people living with psychosis. This presents a unique opportunity for civic engagement, the active involvement of patients, carers and communities in mental health care, to shape emergent services. In-depth explorations of the views of professionals and other key stakeholders in mental health care on the use of civic engagement in Indonesia are lacking which contributes to a limited understanding of its potential in this regard. The study aimed to explore contemporary professionals’ and other key stakeholders’ perspectives on the current use of and potential for civic engagement to strengthen mental health systems in Indonesia.

**Methods:**

Qualitative interviews were undertaken and analysed using thematic analysis underpinned by a critical realist approach. Eighteen multi-disciplinary professionals and lay health workers involved in mental health care in Jakarta and Bogor and 10 national key stakeholders were recruited.

**Results:**

Despite high levels of awareness of and support for civic engagement amongst mental health professionals and policy makers combined with a nascent grass roots movement, analysis revealed unstructured and insufficient mechanisms for civic engagement which resulted in ad-hoc and mostly superficial levels of involvement activity. Civic engagement was thought to require a marked shift in existing practices as well as organisational and societal cultures. Challenging stigma is a key feature of civic engagement and our analysis highlights the relevance of social contact methods which are locally and culturally contextualised in this regard. Our findings point to a need to expand current definitions of civic engagement which focus on indivdiual enablement to ones that also encompass environmental and organisational enablement to optimise the future use of civic engagement in mental health settings.

**Conclusions:**

Key mental health stakeholders have identified that central aspects of Indonesian culture are well aligned to the ethos of civic engagement which has the potential to facilitate the enactment of recent global health policy. However, full realisation is likely to be impeded by prevailing paternalistic cultures in mental health services and high levels of stigma and discrimination towards those with mental illness in Indonesia without intervention.

## Background

Service improvement in global mental health services is a key focus of policy and practice mandates in an attempt to improve care for people living with psychotic disorders such as Schizophrenia [1]. This is of particular salience in low-and-middle-income countries (LMICs) due to limited financial and human resources, which have contributed to treatment gaps in excess of 90% in some countries [2]. There is a well-established translational gap between intervention development and successful implementation [3, 4]. This has been identified as an area for particular attention for service improvements. However, the implementation of novel interventions within low-and-middle-income countries remains poorly understood. Here, we examine the potential use of civic engagement to strengthen Indonesian mental health services from the perspectives of professionals and key stakeholders to progress an understanding of the barriers and enablers to implementation.

Indonesia is an archipelago in South East Asia and the fourth most populated country in the world [5]. Indonesia is struggling to meet the needs of people with psychosis and their families with treatment gaps in excess of 90%. This contributes to significant mortality and morbidity associated with psychosis compared to other countries in the world [6]. The physical restraint (known as ‘pasung’) of people with mental health problems in health and community settings is common in Indonesia as in other developing countries [7]. This type of restraint which includes securing people to fixed objects or locking them in confined spaces can be brief and sporadic or last for a number of years [7–9]. Low awareness of mental health problems, patients’ aggressive or violent behaviour, unemployment in the family, inaccessible or unaffordable care and stigma contribute to the use of pasung [10, 11]. Mental health is now a national priority and Indonesia has emphasised their formal commitment to improving human rights by joining the United Nations Human Rights Council and the United Nations Security Council, making important changes to domestic law and mandating minimum standards of care [10]. Such legislative improvements provide a unique opportunity for civic engagement initiatives to shape emerging mental health systems and ensure they meet the needs of the people they aim to serve.

Public participation in relation to health system strengthening has been a central feature of global health policy over the last thirty years [12]. It is considered an important part of health promotion and community development and is supported by a substantial body of evidence [13]. The World Health Organization recently endorsed civic engagement as a global strategy to improve health through the provision of more person-centred care [14, 15]. Civic engagement has been defined as ‘a process by which people become actively and genuinely involved in defining the issues of concern to them, in making decisions about factors that affect their lives, in formulating and implementing policies, in planning, developing and delivering services and in taking action to active change’ [16]. In low-and-middle-income countries in particular civic engagement has been proposed as an important mechanism through which mental health systems could be strengthened [17, 18]. In a mental health context, civic engagement is an umbrella term which encompasses a wide range of activities which share a central recognition of lived experience as a form of expert knowledge which should considered alongside and held in the same regard as other forms of knowledge [19]. Activities include shared decision making, service user movements to influence policy and change health services, service user led research and patient and public involvement in the design and delivery of mental health care [19, 20].

Drivers of civic engagement has been mostly associated with the survivor/user movement and the work of grass roots community organisations [21]. Civic engagement has particular salience in a mental health context given the historical culture of health services as being associated with surveillance and monitoring and because health services have the power to detain and treat people against their will under mental health legislation where this exists [22–24]. There is encouraging evidence demonstrating the effectiveness of civic engagement at multiple levels within the health system [25, 26]; however, recent evidence indicates a persistent failure to implement such principles successfully and consistently within mental health services [27–30].

Implementation theory provides a number of possible explanations for this translational gap including negative organisational influence [31], lack of resources [32, 33] and professional resistance to civic engagement [34]. Current evidence suggests that focusing on understanding local contexts and interactional processes may yield better understanding of implementation challenges than relying on macro-level theories which have hitherto be unable to identify generalisable strategies for organisational change [35]. Recent systematic reviews in other low-and-middle-income countries have further highlighted the importance of understanding local contexts prior to implementation [36]. For example, the ‘Emerald’ project which aims to develop a scalable model of patient involvement in six low-and-middle-income countries (Ethiopia, India, Nepal, South Africa, Nigeria and Uganda) conducted qualitative research underpinned by phenomenology in each country which identified important contextual barriers specific to individual country that required targeting during intervention development and implementation [29, 30]. Interestingly, existing evidence has tended to focus on the barriers to implementation to the detriment of potential facilitators likely to be important in developing a holistic understanding of civic engagement enactment [37].

Rigorous qualitative studies exploring the potential use of civic engagement to strengthen Indonesian mental health from the perspective of health professionals and national stakeholders have not been undertaken. Research undertaken with patients and carers point to the potential utility of such approaches [32]; however, given that the majority of care for people with psychosis is provided by mental health nurses and psychiatrists in Indonesia it is imperative that their views are considered to ensure optimal implementation.

### Aims

To explore the perspectives of contemporary professionals and other key stakeholders implicated in mental health services in relation to the role of civic engagement to strengthen mental health systems in Indonesia.

## Methods

### Study design

Given the exploratory nature of the study, we used a qualitative approach incorporating individual interviews. This study was part of a larger programme of work to explore the potential implementation of civic engagement within Indonesian mental health services [38]. The views of patients and carers are presented elsewhere [32]. A video documenting wider study activities can be found here: https://www.youtube.com/watch?v=aYdX0FPvtOY

### Participants

Health professionals were recruited from two study sites in Indonesia; Jakarta and Bogor. National key stakeholders who were considered to hold national policy and decision making roles in mental health services in Indonesia were also invited to take part in the study. Both front line professionals and national key stakeholders were recruited to the study in order to ensure that a range of perspectives were elicited in relation to the use of civic engagement within Indonesian mental health services. The former were considered likely to have a deep understanding of the issues facing professionals delivering health care on the ground whilst the latter were thought to be able to illuminate issues relating to national policy and practice initiatives. Previous studies have found that data are required from both types of participant in order to gain a full picture of the potential implementation of interventions. For example, front line professionals have been found to focus on inner setting features (e.g. characteristics of the implementing organisation) with national key stakeholders more likely to consider outer setting features (e.g. economic, political and social contexts) [24, 39]. Consideration of both inner and outer setting features of implementation contexts is fundamental to developing an in-depth understanding of the potential use of civic engagement within Indonesian mental health services.

Recruitment strategies for front line health professionals included email invitations from the study team to all eligible participants in study sites. People were eligible if they had experience of working with people with psychosis in either Jakarta or Bogor. We used purposive sampling to ensure there was equal representation from different occupational roles. See Table 1 for further information on study participants. National key stakeholders were identified from a database compiled by the research team of key people in relation to policy making and mental health decision making nationally in Indonesia. This database was supplemented by analysis of a survey undertaken in Jakarta and Bogor of all mental health professionals where respondents were asked to identify the most powerful and influential people in mental health in Indonesia [38]. Participants were selected and recruited based on their knowledge positions and the extent to which they were considered immersed in the critical understanding of contemporary mental health services and the wider political landscape in Indonesia.

**Table 1:** Information on study participants

ID Number	Profession	Gender
102	Psychologist	M
103	Psychiatrist	M
104	Psychiatrist	F
105	Psychiatrist	M
106	Psychiatrist	F
107	National Key Stakeholder	F
108	National Key Stakeholder	F
109	Nurse	F
110	Nurse	M
111	Social worker	F
112	Nurse	F
113	National Key Stakeholder/nurse	F
116	Social worker	F
117	National Key Stakeholder	F
118	National Key Stakeholder/nurse	F
119	National Key Stakeholder	M
201	National Key Stakeholder/psychiatrist	F
202	Psychiatrist	M
203	National Key Stakeholder/psychologist	M
204	National Key Stakeholder/social worker	F
205	National Key Stakeholder/Psychologist	F
206	Kader	F
207	Kader	F
208	Kader	F
209	Kader	F
215	Nurse	M
216	Nurse	M
217	Nurse	F

Interested parties contacted a member of the research team to discuss participation. During this discussion eligibility was assessed by researchers along with availability for interview and preference in relation to face-to-face or telephone interviews. All potential participants were sent an information sheet prior to the interview and given the opportunity to ask questions about the study in order for them to make a fully informed decision about participation. All participants provided written consent prior to the commencement of interviews.

### Data gathering

A total of 28 interviews were carried out in Bahasa Indonesian between August 2018 and June 2019 by HS, II, SI1, GH and SI2 (male and female academics at the Ministry of Health or the University of Indonesia with a PhD and significant post-doctoral qualitative experience). Eighteen interviews were undertaken with health professionals and workers that support health professionals (e.g. kaders – trained community volunteers). All participants had direct experience of working with people with psychosis in Jakarta and Bogor. Ten interviews were carried out with national key stakeholders who had experience of directly inputting into decision making related to mental health services. Participants were offered the choice of participating in a face-to-face interview in a private room at their workplace (*n* = 23) or a telephone interview (*n* = 5). The duration of interviews was between 35 and 60 min. Some participants were known to the researchers prior to participation and participants were aware of the rationale for undertaking the study as this was documented in the participant information sheet.

Previous research has demonstrated a sustained failure to effectively implement civic engagement principles within mental health services [27–30]. Interview schedules were designed to examine current conceptualisations of civic engagement in Indonesian cultures and to explore the potential involvement of service users, family members and communities in the design and delivery of mental health services in Indonesia with a view to developing a civic engagement framework for future use in Indonesia to strengthen mental health systems. Interview schedules specifically covered current perspectives on the involvement of patients, carers and communities in Indonesian mental health services and the potential use of civic engagement to strengthen developing mental health services in Indonesia. This included an exploration of potential facilitators and barriers to implementation at a micro (between individuals), meso (service or community level) and macro (national) level.

All interviews were digitally audio-recorded, transcribed verbatim, anonymised and translated into English to support analysis by a member of the research team. In line with guidelines for qualitative research, 5% of transcripts were back translated using an independent translation company to ensure accuracy of translation [40]. No concerns were raised when back translations were compared to the original transcripts.

Data saturation is a widely accepted principle in qualitative research, which is used to determine when data collection can be stopped [41, 42]. Data saturation was a standing item on research team meeting agendas with the whole team discussing emerging findings from completed interviews and any new information that was arising from additional interviews. Data collection continued until consensus was reached amongst the research team in team meetings that sufficient data had been collected to address the research questions and that no new information was arising in interviews (*n* = 25). Three further interviews were undertaken to ensure that further data collection was not necessary. Data saturation in multi-site studies has been found to occur between 16 and 40 interviews and our sample size of 28 falls within these parameters [43].

### Data analysis

Transcripts were anonymised at the point of transcription by members of the research team and analysed using thematic analysis [44] supported by NVivo. Analysis was underpinned by a critical realist epistemology, which asserts that knowledge can be shared between groups of people but each person’s understanding will be affected by their own individual beliefs [45]. This combination of thematic analysis and a critical realist epistemology allowed for a deeper level of analysis to be undertaken [44].

The thematic analysis consisted of a number of stages. First, transcripts were read and reread to ensure familiarisation with the data. II, KJ and HB then independently coded the first 8 transcripts before meeting to agree a final set of codes. During this meeting, analysts identified and discussed any discrepancies in coding, removed duplicate codes and combined similar ones before organising codes into overarching themes and agreeing a preliminary thematic framework. This framework was then applied to the remaining transcripts by II with minor additional changes made to the framework during this process. Finally, the framework was shared with the wider study team which included health professionals and people with lived expeirence to allow consensus to be reached that the framework reflected the dataset as a whole. The final stage of analysis was to draft the manuscript using detailed descriptions of identified themes supported by segments of raw data to illustrate interpretations.

### Reducing bias

Members of the research team met regularly to ensure data analysis remained grounded in the data and the experience of professionals working in mental health in Indonesia. The preliminary analysis was also presented to the study advisory group (12 people who either had lived experience of psychosis or cared for someone with a diagnosis of psychosis) at a meeting in November 2018. Their feedback contributed to the analytical process through checking of interpretations and theme development.

### Ethical approval

Ethical approval was obtained from the University of Liverpool Research ethics committee (Ref: 2715) and the Faculty of Nursing Research Ethics Committee, University of Indonesia (Ref: No. 115/UN2.F12.D/HKP.02.03/2018).

## Results

Themes were interpreted from the data which were considered to constitute an in-depth understanding of the current use of and potential for civic engagement to strengthen Indonesian mental health services from the point of view of professionals and national key stakeholders. Identified themes are presented in sections, which relate to the main topic areas covered in interview schedules (e.g. Current understanding and use of civic engagement in Indonesian mental health services and The feasibility of civic engagement within Indonesian mental health services: barriers and facilitators to future implementation). The third section represents a theme in its own right and codes that were used to develop this theme were found across topic areas.

Professionals and key stakeholders had similar views about the use of civic engagement but quotes provided identify each participant’s role for transparency. Six of the 10 national key stakeholders were also professionally trained and their background is presented along with the quote.

### Current understanding and use of civic engagement in Indonesian mental health services

#### High levels of awareness but limited application of civic engagement in Indonesian mental health services

Participants reported high levels of awareness of civic engagement and of the potential benefits to Indonesian health services. Professionals provided detailed accounts of their understanding of civic engagement which included the active participation of patients, carers and communities in the promotion of mental health as well as the design and delivery of mental health care. In line with other research in South East Asia, conceptionalisations of civic engagement extended to a societal level rather than an individual level more commonly associated with western models of involvement and engagement [32, 46]. The strength and consistency of these perceptions was noticeable and is likely to reflect the centrality of the concept in current global health policy rhetoric.

*For me personally, civic engagement has to be in accordance to the needs of the public, because if the public is not engaged in the making of these policies, they will become useless as it will not be in accordance with the public’s own needs.* ***National Key Stakeholder/*** **Psychiatrist - 201**

*If the patient was married, so his partner will be our priority or maybe a certain family member in a family. So if he has a family, it is how we empower his partner because his partner will be the one who spends the longest time with the patient. And next, is his family. Could be his parents, his siblings, or any significant others in that family that has time, chance, ability, and willingness to engage.* ***National Key Stakeholder/*** **Psychiatric Nurse - 118**

Community-based health volunteers (kaders) were considered by participants to be a good example of a civic engagement activity in Indonesia and it was felt that there was scope for this lay workforce to undertake further civic engagement activities.

*A good thing about Bogor is that we have kader, who are well informed and educated from the health service or social workers. Other than health kader, we have community kader, public figures, religious figures, they even come with the patient to primary health service. I think that is awesome.* **Psychiatrist - 106**

*Maybe the kader could be trained about how to take care of them in the house so then they can teach the family because it is important to know it, don’t only rely on the health worker to come visit the patient but kader should be trained and family will come to the kader first, so kader can handle it if something happens.* **National Key Stakeholder - 107**

Examples of other activities that participants considered well aligned to the principles of civic engagement can be found in Table 2. Identified activities ranged in the scale of involvement of patients, family members and community members from relatively low level (e.g. leading recreational activities or being involved in activities that are health professional led rather than co-designed or co-produced with people with lived experience of mental illness) to more meaningful levels of involvement (e.g. involvement in the development of the Mental Health Act or shared decision making about treatment plans).

**Table 2:** Examples of current civic engagement activities in Indonesia as reported by study participants

ID 201	*In mental health we have involved them [people with lived experience of mental health problems] when constructing the Mental Health Act, and for the Narcotics Act also we had drug users involved and others too.*
ID 102	*So the model we follow is meaningful youth participation. For example if there is a panel about mental health for teenagers that panel should include a teenager, not only the old experts, because [without this] there is a possibility that suggestions made will be biased and irrelevant to today’s circumstances. For example young people can give suggestions for improvements to law, research, questionnaires, and also the improvement of interventions, that is what we try to do in our community.*
ID 116	*As I’ve said, our organization is an advocate group, and this advocation is done by people with mental health problems, so we don’t only accept programs but we also criticize, suggest, make programs better, and advocate for programs that are needed.*
ID	*People from the community and us as an organization are advocating seriously to the health authority, and recently the health authority decided to register every person with mental illness, including ones with chronic mental illnesses.*
ID103	*What already happen here is especially in a community like KPSI (Komunitas Peduli Skizofrenia Indonesia/Indonesian Schizophrenic Care Community), Bipolar Group, Substance Abuse Group, and we once asked them to discuss about mental health service like mental health that caused a physical illness like HIV/AIDS, chronic kidney disease, we asked about their hopes and merged it with other stakeholders and psychiatric hospital parters’ hopes in a discussion … … yes, we invite the user too, and stakeholder, partners, citizens, KPSI, Bipolar Care Group, Substance Abuse Group, and a group of people with chronic physical disease. Actually since 2009, we held it every 5 years. For RSMM (Marzuki Mahdi Psychiatric Hospital), we always connected with them to improve our services.*
ID203	*We can see it in communities of people who have experienced it. For example, people with bipolar disorder or schizophrenia would have their own community, and they would engage with people who are healthy to spread awareness and ask for support. So there are now bipolar, schizophrenia, autism communities, those are the concrete examples that the society has done.*
ID104	*In Pacitan, it’s Head is very concern with Pasung so there is a community of patients who have experienced Pasung and they become merchants in the market, every month they have a gathering, traveling together to the beach … .. There was a cooperation with Traditional Medicine Company and we helped it with providing a field and they teach and train them to grow vary of medicinal plants and sell the harvests to the company.*
ID105	*When planning management, I tried to involve family and patients. Sometimes patients are accompanied by Kader, or care givers who are not close relatives. We try to make a plan or I like to call it road to recovery for this patient … … I think the patient is ready to go home then we do discharge planning, and that’s where the patient and family get involved to see what helps recovery to get better when they go home.* *For example there is Ms. X who often teaches handicrafts, she will say “can I take the session?” Then I say “oh of course, we can set the time” (105)* *There are also families who do have family members with mental health problems, then want to teach others. So he is a caregiver, but he volunteers for others.*
ID111	*There are some custom topics family can choose to adjust with their problems so then there is an engagement from family because they have their own rights, we are not always “feeding” them. And then we also trained them to make their own rules and schedule for patient’s daily activity.*
ID109	*I’m a clinician and I have a direct connection with patient. So when I give nursing care, I’m not just judging for what I’ve found, but I will explore deeper what’s in the family, and make my decision for the right treatment, together designing the nursing planning with patient and family. So we engage them, the patient and family.*

#### Perceived benefits of civic engagement for Indonesian mental health services

When critically considering the use of civic engagement to strengthen mental health systems, participants demonstrated a propensity to focus on the benefits with significantly less consideration given to potential risks. This contrasts with professionals from the Global North who tend to resist involvement activities because of concerns around risk management and capacity [47]. Identified benefits included those at an individual level such as reduced duration of untreated psychosis, increased confidence and motivation amongst service users which was related to increased likelihood for recovery and those at a health service level through increased efficiency and more patient centred care.

*The effect could be very positive. Patients will get their motivation and support to heal. For us, as health workers, we get positive effects too because engagement is very helpful for our program, it will be easier if we do it together to reach our goal.* **National Key Stakeholder - 107**

*As a service provider, like me, civic engagement could decrease the duration of untreated psychosis. So it will take a shorter time to heal them and patients will have a better prognosis if they come to the health facility as soon as possible. And it will also decrease the treatment gap of a medication in some places, that’s the most important thing. Other than that, to maintain the patient’s health.* **Psychiatrist - 106**

An additional benefit identified by participants was the reduction of stigma associated with mental health diagnoses which was considered pervasive in Indonesia. Participants felt that involving patients, family and communities in mental health care could increase societal awareness of mental health and break the cycle of stigma and discrimination. In particular, human rights violations such as the confinement and detainment of people with psychosis in institutions or family homes against their will, (‘pasung’ in Bahasa Indonesian), could be reduced or even eliminated through increased use of civic engagement [10, 48].

*The challenge now is stigma and discrimination. These are the real issues to tackle, to make sure that mental health problems are not marginalised. This is the real challenge that we want to bring forward, and it can only happen if the public is involved. Eventually this is what will stop the chain of stigma and discrimination, and people can be actively involved to educate or give information for others.* ***National Key Stakeholder/*** **Psychiatrist - 203**

*There is still so much kind of stigma out there, and I’m sure with their [families] understanding, they will know how to take care of the patient, and they will engage in that process, so we hope this stigma will decrease slowly.* **National Key Stakeholder – 108**

#### The role of paternalistic cultures in undermining the ethos of civic engagement

Despite this surface level value attributed to civic engagement, the actual implementation of civic engagement in Indonesian mental health services was diminished by pervasive paternalistic cultures within services when narratives were examined in detail. Participants felt that such cultures were entrenched in contemporary mental health services in Indonesia.

*I see treatments in both hospitals and institutions being more assumptive, or not involving the patients enough in their treatments. This is more the case in social institutions that are privately managed. They still assume that they have to take care of everything for the patients, that they are the ones who know everything, and this is something that inhibits [active participation/civic engagement].* **Psychiatrist – 202**

*I think it [civic engagement] is not working as we expected because designing mental health service is [still] only done by health workers, you see it still one way.* **Psychiatric Nurse - 110**

Interestingly, professionals took limited responsibility themselves for enacting civic engagement in mental health services and often blamed patients for a perceived lack of involvement. Some professionals felt that that patients and family members should take responsibility for self-care and be able to challenge decisions made by professionals and identified a need to empower people to be able to do this.

*Other than knowledge and awareness, they also have to have willingness to decide their choice in order to get treatment until they can take care of themselves. The whole point is the patient has to develop a skill to take care of himself.* **Psychiatric Nurse - 112**

*The obstacle from patients and families is the level of their education. For those who have low educational level, we will find it is difficult to involve them further in determining what will be done in the future.* **Psychiatrist - 105**

A small number of professionals focussed on increased compliance from patients and family members as a benefit of civic engagement which contravenes the ethos of civic engagement. Primary drivers for involvement agendas relate to the advocation of the personal and collective rights of people who use services rather than complying with treatment mandated by health services [49].

*I think working together with patients and their family members will increase their compliance as well as the family's trust, so that cases of dropping out of treatment or relapsing as a result of a stressor that may happen during the treatment will decrease.* **Psychiatrist - 202**

### Feasibility of the use of civic engagement in Indonesian mental health services: barriers and facilitatiors to future implementation

#### The alignment of civic engagement to central features of Indonesian culture

There was consensus amongst participants that central aspects of Indonesian culture were well aligned to the ethos of civic engagement which had the potential to facilitate its use in mental health services. In particular, civic engagement was consistent with the ‘Pancasila’or foundational philosophical principles in Indonesia; belief in God, a just and fair society, a unified Indonesia, democracy and social justice. Family relationships and community cohesion more generally were considered stronger in Indonesia when compared to Western cultures.

*The culture of Indonesia is very humane. We have Pancasila (foundational philosophy of Indonesia), the opening of the constitution. Article 28H and others, it's already regulated by the government, by the society. So people who don't care are not Indonesians, that means that they don't have a religion, they don't have human values.* **National Key Stakeholder - 119**

*I think there's potential in the society. We have the culture of ‘gotong royong’ (communities working together), that's a strength of our society. We have trust among ourselves; we have a culture of compassion, to help others, to donate. We can better ourselves with this. I hope there would be a good governance, that the governments are involved, that the regulations are enforced.* **National Key Stakeholder/ Social Worker - 204**

*In Indonesia, familial relationship are a lot better than in western countries, and especially we have better social lives [as communities], therefore if they are well-informed they actually have a good potential to encourage themselves, their family members, and the community around them to be engaged too.* **Psychiatrist - 202**

Participants described a shared desire among patients and family members to learn more about mental health, which could further facilitate the implementation of civic engagement in Indonesia.

*They enthusiastically join every time we have a mental health program they will come and participate in the event. Usually we do education, sharing session, in hospital and in community or local health center, and there will be about 20 to 30 of them who come. In small group activity like a specific specialty, for example for the patient’s family, it will be a small group. But for a common training we’ve done it a lot. Their enthusiasm is very good and they want to be involved.* **Psychiatric Nurse - 217**

#### The importance of changes to international and domestic law and the role of third sector organisations to support the implementation of civic engagement

At a macro level, participants described how recent changes to international covenants and domestic law (e.g. the Disability Act and 2014 Mental Health Act) had improved protections of human rights for people in Indonesia. Mental health care provision itself had improved recently since the prioritisation of mental health at a national level through the development of mental health indicators which had led to the provision of basic community mental health care. These improvements have been accompanied by a growing but disparate user movement in the form of increasingly active community organisations which has the potential to provide further support for the use of civic engagement in Indonesian mental health services. Such organisations are increasing awareness of mental illness in communities, providing direct support for individuals, family and communities in relation to mental health, advocating at a health service level for better care and campaigning for the rights of people with mental health problems at a national level (Table 2).

*If we examine public policies that encourage civic engagement, we can see, for example, the Disability Act. Now they have the same rights [as any other people] so they're encouraged to be more active in their social lives, and eventually this will encourage them to be involved in clinical decisions. We also have the Mental Health Act that emphasises how important it is to protect them, their rights, and their finances so that they can manage their own finances and their own rights.* **Psychiatrist - 202**

*In the Ministry of Health we have 12 main indicators of a healthy family (indicator keluarga sehat – indicators of healthy families) which include mental health. In this case, the Ministry of Health and the Ministry of Social are responsible and they are very supporting this so I think there are so many policies that make civic engagement a priority* **Psychiatrist - 104**

*We have PMK (Peraturan Menteri Keuangan/Ministry of Economy’s constitution) number 35 that said mental health should be treated and it’s already in SPM (Standard Minimum Pelayanan/Service Minimum Standard – minimum standards of care) of every regions and cities, that policy.* **Psychiatric Nurse - 109**

#### Low levels of awareness and desire for civic engagement within local communities

Participants identified a number of barriers at an individual level to the optimal use of civic engagement. These were mostly attributed to patients and included a lack of knowledge about mental health generally, patients lacking insight into their condition or not having the requisite capacity to be involved. For example, professionals coalesced in their views that patients may not be able to participate because of their condition due to symptom manifestation or side effects of drug treatment. Others felt that such perceptions represented ‘self-stigma’ on the part of the patients and family members rather than actual deficits that would prohibit involvement. These factors contribute to low desire for and expectations of civic engagement, and reduced ability to be involved in mental health services.

*The obstacles? Maybe a patient with mental illness usually runs out of energy so it will be hard for him to participate more if his condition is not good enough.* **Psychologist - 102**

*And this also happens inside the patients themselves, feeling self-stigma that it's true I'm useless, I'm incompetent, I can't make the right decisions; if they're being given a chance they'll leave it to the doctors, unable to make decisions themselves.* **Psychiatrist - 202**

*Their willingness to engage is still passive, not all of them that have a family member with mental illness feel that they have a right to contribute in.* **National Key Stakeholder/Psychiatric Nurse - 118**

#### Lack of local implementation strategies and regulatory procedures

Despite the aforementioned improvements to national policy and laws related to the treatment of people with mental health, there was no guidance about local implementation or regulations to ensure compliance with these mandates. Participants also described competing laws and regulations, which are openly discriminatory against people with mental health problems and detract from these new guidelines. Identified examples included people being subject to mental health screening in order to attend school or apply for jobs and a national insurance policy which does not cover health problems related to drug abuse and suicide.

*The regulation which requires mental health screening in order for people to work or enroll in schools, I think this hampers their engagement as it would be difficult for them to get a formal job or be in school.* **Psychiatrist - 202**

*Mental Health Act has been achieved in 2014. Now it's time to fight for the implementation. Its derivatives aren't finished yet and I think the government needs to do that.* **National Key Stakeholder/Psychologist – 205**

*It’s hard for us to advocate and it’s not gonna succeed in only one time or two times advocating procedure. Social Office has so many programs, local health center has so many workers, so many nurses handle tuberculosis, etc. But in mental health issue, it will be just an option, a local health center can choose whether they want to have that facility or not.* **Social Worker - 111**

#### Parity of esteem in relation to awareness about and resources for mental health

Participants felt that Indonesia was still a long way from achieving parity of esteem between mental and physical health conditions. Insufficient resources for rehabilitation, social support programs, building infrastructure or staff training were further barriers to the successful implementation of civic engagement in Indonesian mental health services. These issues were further compounded by low levels of mental health awareness amongst senior policy makers, poor coordination between government agencies and insufficient mental health leadership at a national level.

*For now, it will be budget. Because if we want the public to make a move, or we want to educate them, it will cost much money, a well-arranged program. The information is the only thing we can prepare. And the education media, we will need it too.* **Psychiatrist - 106**

*For me, the emphasis of the problem is on the weakness of leadership. So if leadership in mental health is still slow like that, the developing of mental health will be very slow too. Especially if we think about the civic engagement.* **Psychiatrist - 105**

*One of the quality indicators is integration, inter-collaboration that isn't only inter-professional, but everything. Lastly, efficiency. This doesn't take place because everyone works separately.* **National Key Stakeholder - 119**

#### The prevalence of stigma and discrimination towards people with mental illness in Indonesia

**Fig. 1:**
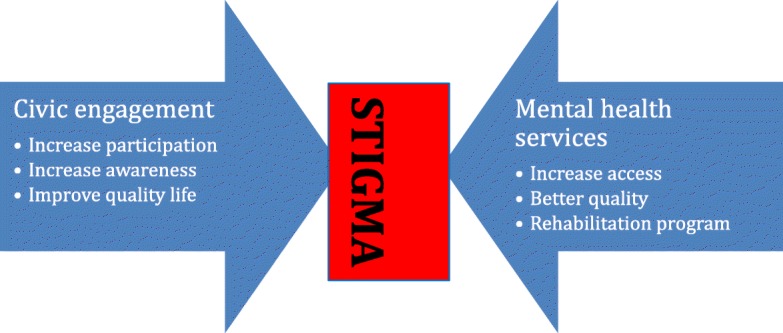
Stigma blocks the synergism between civic engagement and mental health services

Irrespective of professional role, participants held strong views about the need to address stigma towards people with mental illness in Indonesia to enable optimal utilisation of civic engagement. Whilst acknowledging that attitudes towards those with mental illness had improved over recent years, narratives focussed on the significant challenges which persisted. Stigma, both felt and enacted [50], was considered to be deeply entrenched in Indonesian society partially connected to certain cultural beliefs in Indonesia. Stigma and fear represented significant barriers to civic engagement at all levels both within and outside of health services (Fig. 1). Professionals considered such entrenched attitudes and behaviours entrenched and resistant to change.

*I think stigma is the number one factor that hinders civic engagement. Firstly, the stigma that people with mental disorders are people with no hope, can't make decisions, don't know what's best for themselves, can't work or go to school, can't have a family, can't get married, etcetera. This stigma is really damaging and negative, and this stigma exists from the family-level to the community, government, to medical personnel, there are a lot of them who still hold this stigma.* **Social Worker - 116**

*From the eyes of a general public, I think not only that the public doesn't understand, but also there are a lot of myths and stigma [surrounding mental health] that prevent people from participating [in civic engagement] … .. But people are behind not only because they don't have the knowledge, but also our culture that rejects anything different. The stigma, the myth that mental illness is something that happens because you don't have enough religious faith, because they were mistreated, because they got possessed, because they were sinful. This is something that's really happening, it's very prevalent in the society.* **Social Worker - 204**

Felt stigma was considered to result in shame amongst family members and patients which was thought to decrease motivation and confidence to participate in involvement activities. This inhibited patients and family members accessing support from services or people in their communities and exacerbated social exclusion. Enacted stigma was considered to inhibit recovery related activities such as employment and participating in valued activities.

*For example, in communities, there are churches, pastoral counseling, counseling units, shelters. I think there are some community efforts, even if it's not specific to mental health problems. But they don't talk about that, there is stigma associated with going to counseling. When they go to counselors they're asked, Hey, are you stressed? Are you crazy? If their friends come and talk to them like that, they'd cancel their plan to come counseling.* **National Key Stakeholder/Psychiatrist – 205**

*But when they are healed and ready to work, there will be a problem when they have to write about their medical record and they’re confused “should I really write my medical history?” if they write that they ever have a mental condition and was an inpatient, they will be automatically eliminated.* **Psychiatric Nurse – 216**

*The family feels ashamed … I also won't mention to people about the condition of their ill family members.* **Kader - 207**

Despite stigma being the greatest barrier to civic engagement reported by professionals, analysis revealed some promising signals of recent improvements in awareness of and attitudes towards mental illness in Indonesia. Such improvements were attributed to mental health campaigns led by health services and  both governmental and community organisations. Social media was considered an important method by which to effectively reach large numbers of people in Indonesia for relatively little cost. Campaigns included educational activities and encouraging people to use fewer stigmatising terms to describe mental illness. Some hospitals have started inviting members of the public to take part in education programmes and running peer volunteer opportunities.

*For me, this shows that people are now more aware, more open to report their family members. They're no longer ashamed, no longer resistant, are not stigmatised, in that context it's a better awareness … .. I don't think there's a downside, just that at first the challenge would be stigma, political interests, those might be the obstacles. But if it's pushed forward, those obstacles can be turned into something positive.* **National Key Stakeholder/Social Worker - 204**

*For example as a director I give them material in a meeting to discuss disaster management in terms of mental health. I also speak in forums like public education in radio, television, newspaper, I tell my staff to do public education.* **Psychiatrist - 103**

## Discussion

A qualitative study was undertaken to develop an in-depth understanding of professional and national key stakeholders’ views on the potential for civic engagement to strengthen Indonesian mental health systems to further understand barriers and facilitators to implementation. Data from the current study has identified an important disconnection between global health policy advocating civic engagement [14] and the capacity for implementation on the ground in Indonesia. This is the first study to explore the use of civic engagement from the perspectives of front-line professionals and national key stakeholders in Indonesia and to identify facilitators to civic engagement as well as the factors contributing to this translational gap which are fundamentally important to those wishing to implement civic engagement both in Indonesia and in other low-and-middle-income countries.

Despite recognition of the potential benefits of civic engagement, participants described only limited current activities which remained ad-hoc and predominantly superficial which coalesced with the experiences of patients and carers [32]. The kader programme which uses lay workers (community volunteers trained and supervised by primary health care staff) to deliver basic health care to local communities was the one example of co-ordinated civic engagement activities in Indonesia. The role of kader was developed initially to support child and maternal health in local communities but more recently has been extended other non-communicable diseases including mental health and is associated with a number of health outcomes [51].

Enacting civic engagement in mental health services is more complicated than in physical health services because of historical socio-political contexts relating to coercion and control [22, 23]. Attention needs to be given to the de-implementation of existing practices in addition to the work required to enact new interventions at a patient, professional and organisation level [52]. Successful implementation at the very least requires a shared understanding of civic engagement activities [25]. The current study demonstrates that professionals coalesced in their understanding of and value attributed to civic engagement. Service users and carers in a related study; however, varied more in their understanding of civic engagement which appeared dependant on exposure to mental health services and mental health activism [32]. In line with findings from other low-and-middle-income countries, patients prioritised access to medication and suitable care over involvement activities [30, 32]. Increasing awareness of civic engagement and increasing desire for such activities amongst all stakeholders represents a key intervention point for health services and data highlights the central role of third sector organisations in this regard. These findings point to the need to expand current definitions of civic engagement. Until now, these have tended to focus on the enablement of individuals to become actively and genuinely involved in activities. Our study suggests a need to consider more holistic definitions which include the enablement of organisations and environments as well as individuals in order to realise optimal levels of civic engagement.

In Indonesia as in other low-and-middle-income countries, felt and enacted stigma towards those with mental health problems is pervasive and entrenched in organisational and societal culture and is considered a significant barrier to implementation [30, 53, 54]. This is compounded by limited awareness of human rights, finite resources and misconceptions about mental illness [32]. The World Health Organisation did not identify stigma as a priority area in their recent strategy on people-centred and integrated services [14]. This is likely to be because the strategy did not specifically focus on mental health services. In the current study, stigma was perceived to contravene the potential synergy between civic engagement and mental health service development at all levels within the system (Fig. 1). Research in high income countries has identified stigma as an important barrier to civic engagement [55–57]. However, in line with research from other low-and-middle income countries, the level and extent of stigma in the current study exceeds those identified in higher income countries [30].

The study contributes to existing literature by identifying strategies to challenge stigma. For example, civic engagement was considered well aligned to Indonesian culture on philosophical grounds. In particular, civic engagment was consistent with Pillars 3 and 5 of the ‘Pancasila’or foundational principles in Indonesia. As a result of these principles, family relationships and community cohesion more generally were considered stronger in Indonesia when compared to Western cultures and should be considered an important resource in challenging stigma. Additionally, in order to optimise anti-stigma programmes in low-and-middle-income countries, attention should be given to potential philosophical synergies between cultural values and how these can be used to target interventions. Campaigns underpinned by socio-psychological theories of cognitive dissonance could highlight that Indonesian egalitarian cultural values are inconsistent with negative views about people with psychosis in an attempt to reduce stigma. Such methods have been successful in reducing stigmatising attitudes in other populations [58]. In contrast with European countries, patients and their families should be considered as one unit and attempts to reduce stigma and promote civic engagement should empower individuals at a community level. There is promising evidence on the use of community engagement events incorporating arts-based activities to reduce stigma through increased intergroup contact and enhanced knowledge about mental illness [59–61]. This study points to the need to ensure these social contact methods are locally contextualised for use in Indonesia.

The findings emphasised the significance of legal and policy frameworks to the implementation of civic engagement at multiple levels within the system. Important facilitators at a macro level were identified which are likely to be important to emerging civic engagement activities. Recent law, regulations and policies related to mental health at a national level are now considered to provide an adequate framework for human rights protections [10]. More recently the governmental programme of universal health coverage which encompasses the majority of mental illnesses should promote access to care. Such developments are aligned to recommendations made by the WHO [14]. Mental health is also now considered an important local priority as mental health is one of the 12 indicators considered the responsibility of puskesmas or primary care centres in Indonesia [62]. However, the study also identified legacy policies which directly contravene the ethos of civic engagement. What is currently lacking are implementation plans at a local level (Desa and kelurahan - urban and rural village level) likely to be fundamental for optimal implementation and routinisation of civic engagement activities in practice and to enable legacy policies not considered in line with civic engagement to be circumvented.

In line with the views of service users and carers, participants in the current study highlighted the significant role of third sector organisations to promote civic engagement in mental health at multiple levels within the system [32]. This included increasing desire for involvement amongst communities, reducing stigma and advocating for macro level changes. For example, Indonesia’s mental health law was first enacted in 1967, but then amalgamated into the general health law in 1996. After years of advocating by various organisations the mental health law was re-enacted in 2014. Following this enactment, mental health law, health programs and policy on mental health have improved significantly demonstrating the power of third sector organisations once mobilised. At the community level, third sector and non-government organisations are considered best placed to deliver holistic care and undertake civic engagement, especially in relation to increasing awareness and challenging stigma [32], which adds further support to their critical role in future implementation.

### Strengths and limitations

This study draws its strength from the in-depth nature of data collection from a wide range stakeholders with a breadth of experience. Such methods of data collection allow participants to raise concerns of particular salience to local contexts which would not have been possible through the more restrictive collection of data through questionnaires. Professional views about civic engagement in low-and-middle-income countries are important to implementation but under-represented in current literature. We have presented the views of service users and carers elsewhere [32].

Our combination of thematic analysis and a critical realist epistemology allowed for a deeper level of analysis to be undertaken which examined the individual, organisational and cultural influences on involving people in mental health care. We did not observe any interactions between health professionals and patients or any civic engagement activities. Future research should consider such approaches (e.g. clinical observations or ethnographical methods) to build on the current data. Data was collected from 18 professionals from two sites in Indonesia: Jakarta and Bogor and 10 professionals involved in national level decision making about mental health. Future studies could include recruitment from other geographical areas in Indonesia and consider including cultural and religious leaders to supplement this data presented here.

## Conclusion

Although mandated in global health policy for low-and-middle-income countries and acceptable in principle, optimal use of civic engagement in Indonesia is lacking. Full realisation of involvement and engagement agendas requires an in-depth understanding of the cultural and societal contexts in which health provision occurs prior to intervention development and implementation.

## Data Availability

The datasets generated and analysed during the current study are not publicly available due to ethical restrictions but are available from the corresponding author on reasonable request.

## Abbreviations

LMIC: Low and middle income countries
WHO: World Health Organisation
UK: United Kingdom

## References

[1] World Health Organisation (2018). WHO Mental Health Gap Action Programme (mhGAP).

[2] Alem A, Kebede D, Fekadu A, Shibre T, Fekadu D, Beyero T (2009). Clinical course and outcome of schizophrenia in a predominantly treatment-naive cohort in rural Ethiopia. Schizophrenia Bull.

[3] Grol R, Grimshaw J (2003). From best evidence to best practice: effective implementation of change in patients’ care. Lancet..

[4] Green LA, Seifert CM (2005). Translation of research into practice: why we can’t “just do it”. J Am Board Fam Pract.

[5] World Population Review (2019). Indonesia Population 2019.

[6] Mathers C, Fat D, Boerma J (2008). The global burden of disease: 2004 update.

[7] Minas H, Diatri H (2008). Pasung: physical restraint and confinement of the mentally ill in the community. Int J Ment Health Syst..

[8] Jorm AF, Griffiths KM, Christensen H, Korten AE, Parslow RA, Rodgers B (2003). Providing information about the effectiveness of treatment options to depressed people in the community: a randomized controlled trial of effects on mental health literacy, help-seeking and symptoms. Psychol Med.

[9] Broch H (2001). The villagers’ reactions towards craziness: an Indonesian example. Transcult Psychiatry.

[10] Irmansyah I, Prasetyo YA, Minas H (2009). Human rights of persons with mental illness in Indonesia: more than legislation is needed. Int J Ment Health Syst..

[11] Laila NH, Mahkota R, Shivalli S, Bantas K, Krianto T (2019). Factors associated with pasung (physical restraint and confinement) of schizophrenia patients in Bogor regency, West Java Province, Indonesia 2017. BMC Psychiatry..

[12] Frankish CJ, Kwan B, Ratner PA, Higgins JW, Larsen C (2002). Challenges of citizen participation in regional health authorities. Soc Sci Med.

[13] Florin P, Wandersman A (1990). An introduction to citizen participation, voluntary organizations, and community-development - insights for empowerment through research. Am J Commun Psychol.

[14] World Health Organisation (2015). WHO global strategy on people-centred and integrated health services: interim report.

[15] Wallcraft J, Amering M, Freidin J, Davar B, Froggatt D, Jafri H (2011). Partnerships for better mental health worldwide: WPA recommendations on best practices in working with service users and family carers. World Psychiatry.

[16] World Health Organisation (2002). Community participation in local health and sustainability development: approaches and techniques.

[17] Saraceno B (2007). Advancing the global mental health agenda. Int J Public Health.

[18] Saraceno B (2007). New knowledge and new hope to people with emerging mental disorders. Early Interv Psychiatry.

[19] Rose D (2008). Service user produced knowledge. J Ment Health.

[20] Trivedi P, Wykes T (2002). From passive subjects to equal partners: qualitative review of user involvement in research. Br J Psychiatry.

[21] Ramon S, Healy B, Renouf N (2007). Recovery from mental illness as an emergent concept and practice in Australia and the UK. Int J Soc Psychiatr.

[22] Szmukler G (2015). Compulsion and “coercion” in mental health care. World Psychiatry.

[23] Gilburt H, Rose D, Slade M (2008). The importance of relationships in mental health care: a qualitative study of service users’ experiences of psychiatric hospital admission in the UK. BMC Health Serv Res.

[24] Brooks H, Sanders C, Lovell K, Fraser C, Rogers A (2015). Re-inventing care planning in mental health: stakeholder accounts of the imagined implementation of a user/carer involved intervention. BMC Health Serv Res.

[25] Bee P, Price O, Baker J, Lovell K (2015). Systematic synthesis of barriers and facilitators to service user-led care planning. Brit J Psychiat.

[26] Gottlieb BH, Gillespie AA (2008). Volunteerism, health, and civic engagement among older adults. Can J Aging.

[27] Grundy AC, Bee P, Meade O, Callaghan P, Beatty S, Olleveant N (2016). Bringing meaning to user involvement in mental health care planning: a qualitative exploration of service user perspectives. J Psychiatr Ment Health Nurs.

[28] Mental Health Council of Australia and Carers Association of Australia (2000). Carers of People with a Mental Illness Project Final Report. Commonwealth Department of Health and Aged Care.

[29] Lempp H, Abayneh S, Gurung D, Kola L, Abdulmalik J, Evans-Lacko S (2018). Service user and caregiver involvement in mental health system strengthening in low- and middle-income countries: a cross-country qualitative study. Epidemiol Psychiatr Sci.

[30] Abayneh S, Lempp H, Alem A, Alemayehu D, Eshetu T, Lund C (2017). Service user involvement in mental health system strengthening in a rural African setting: qualitative study. BMC Psychiatry.

[31] Anthony P, Crawford P (2000). Service user involvement in care planning: the mental health nurse's perspective. J Psychiatr Ment Health Nurs.

[32] Susanti H, James K, Utomo B, Keliat BA, Lovell K, Irmansyah I (2020). Exploring the potential use of patient and public involvement to strengthen Indonesian mental health care for people with psychosis: a qualitative exploration of the views of service users and carers. Health Expect.

[33] Brooks H, Lovell K, Bee P, Fraser C, Molloy C, Rogers A (2019). Implementing an intervention designed to enhance service user involvement in mental health care planning: a qualitative process evaluation. Soc Psychiatry Psychiatr Epidemiol.

[34] Crawford MJ, Aldridge T, Bhui K, Rutter D, Manley C, Weaver T (2003). User involvement in the planning and delivery of mental health services: a cross-sectional survey of service users and providers. Acta Psychiatr Scand.

[35] May C (2006). A rational model for assessing and evaluating complex interventions in health care. BMC Health Serv Res.

[36] Semrau M, Lempp H, Keynejad R, Evans-Lacko S, Mugisha J, Raja S (2016). Service user and caregiver involvement in mental health system strengthening in low- and middle-income countries: systematic review. BMC Health Serv Res.

[37] Puchalski Ritchie LM, Khan S, Moore JE, Timmings C, van Lettow M, Vogel JP (2016). Low- and middle-income countries face many common barriers to implementation of maternal health evidence products. J Clin Epidemiol.

[38] Brooks H, James K, Irmansyah I, Keliat BA, Utomo B, Rose D (2018). Exploring the potential of civic engagement to strengthen mental health systems in Indonesia (IGNITE): a study protocol. Int J Ment Health Syst.

[39] Rogers A, Vassilev I, Pumar MJJ, Todorova E, Portillo MC, Foss C (2015). Meso level influences on long term condition self-management: stakeholder accounts of commonalities and differences across six European countries. BMC Public Health.

[40] Twinn S (1997). An exploratory study examining the influence of translation on the validity and reliability of qualitative data in nursing research. J Adv Nurs.

[41] Fusch PI, Ness LR (2015). Are we there yet? Data saturation in qualitative research. Qual Rep.

[42] Saunders B, Sim J, Kingstone T, Baker S, Waterfield J, Bartlam B (2018). Saturation in qualitative research: exploring its conceptualization and operationalization. Qual Quant.

[43] Hagaman AK, Wutich A (2017). How many interviews are enough to identify Metathemes in multisited and cross-cultural research? Another perspective on guest, Bunce, and Johnson's (2006) landmark study. Field Method.

[44] Braun V, Clarke V (2006). Using thematic analysis in psychology. Qual Res Psychol.

[45] Robson C (2002). Real world research. A resource for social scientists and practitioner-researchers. 2nd edition.

[46] James K, Brooks H, Susanti H, Waddingham J, Irmansyah I, Keliat BA (2020). Implementing civic engagement within mental health services in South East Asia: a systematic review and realist synthesis of current evidence. Int J Ment Health Syst.

[47] Brooks HL, Lovell K, Bee P, Sanders C, Rogers A. Is it time to abandon care planning in mental health services? A qualitative study exploring the views of professionals, service users and carers. Health Expect. 23;(2):377–87. 10.1111/hex.12650 PMC5980609 29144591

[48] Novy Helena R, Daulima C, Yulia WI (2018). The experience of people with mental disorders in social function adaptation after suffering from pasung. Enferm Clin.

[49] Wallcraft J, Bryant M. The Mental Health Service User Movement in England. London: Sainsbury Centre for Mental Health; 2003.

[50] Scrambler G (1998). Stigma and disease: changing paradigms. Lancet..

[51] Surjaningrum ER, Minas H, Jorm AF, Kakuma R (2018). The feasibility of a role for community health workers in integrated mental health care for perinatal depression: a qualitative study from Surabaya, Indonesia. Int J Ment Health Syst.

[52] Norton WE, Kennedy AE, Chambers DA (2017). Studying de-implementation in health: an analysis of funded research grants. Implement Sci.

[53] Saraceno B, van Ommeren M, Batniji R, Cohen A, Gureje O, Mahoney J (2007). Barriers to improvement of mental health services in low-income and middle-income countries. Lancet..

[54] Hartini N, Fardana NA, Ariana AD, Wardana ND (2018). Stigma toward people with mental health problems in Indonesia. Psychol Res Behav Manag.

[55] Valentine SE, Dixon L, Borba CP, Shtasel DL, Marques L (2016). Mental illness stigma and engagement in an implementation trial for cognitive processing therapy at a diverse community health center: a qualitative investigation. Int J Cult Ment Health.

[56] Corrigan P (2004). How stigma interferes with mental health care. Am Psychol.

[57] Pinfold V, Byrne P, Toulmin H (2005). Challenging stigma and discrimination in communities: a focus group study identifying UK mental health service users’ main campaign priorities. Int J Soc Psychiatry.

[58] Ciao AC, Latner JD (2011). Reducing obesity stigma: the effectiveness of cognitive dissonance and social consensus interventions. Obesity (Silver Spring).

[59] Brooks H, Irmansyah I, Susanti H, Utomo B, Prawira B, Iskandar L (2019). Evaluating the acceptability of a co-produced and co-delivered mental health public engagement festival: mental health matters, Jakarta, Indonesia. Res Involv Engagem.

[60] Hanrahan C (2013). Critical social theory and the politics of narrative in the mental health professions: the mental health film festival as an emerging postmodern praxis. Brit J Soc Work.

[61] Quinn N, Shulman A, Knifton L, Byrne P (2011). The impact of a national mental health arts and film festival on stigma and recovery. Acta Psychiatr Scand.

[62] Astuti TS (2019). Program Indonesia Sehat dengan pendekatan keluarga: analisis kesiapan biaya untuk hipertensi, diabetes melitus, dan gangguan jiwa di Kota Depok 2018–2020.

